# Avacopan-Associated Hepatotoxicity: Clinical Signal, Liver Injury Patterns, and Plausible Mechanisms

**DOI:** 10.7759/cureus.109674

**Published:** 2026-05-26

**Authors:** Luis Guillermo Herrera-Jiménez, Sebastián Arguedas-Chacón, Jeaustin Mora-Jiménez, Esteban Zavaleta-Monestel

**Affiliations:** 1 Department of Pharmacy, University of Costa Rica, San José, CRI; 2 Department of Pharmacy, Hospital Clinica Biblica, San José, CRI; 3 Department of Research, Hospital Clinica Biblica, San José, CRI

**Keywords:** anca-associated vasculitis, avacopan, cholestatic hepatitis, complement c5a receptor, dili, drug-induced liver injury, hepatotoxicity

## Abstract

Avacopan, an oral selective C5a receptor antagonist used as a glucocorticoid-sparing adjunct in severe active antineutrophil cytoplasmic antibody (ANCA)-associated vasculitis, has been linked to a potential hepatobiliary safety concern. This critical narrative review summarizes clinical, real-world, pharmacovigilance, and regulatory evidence on avacopan-associated liver injury, with emphasis on reported injury patterns and plausible mechanisms. The review was informed by a targeted bibliographic search of PubMed/MEDLINE (Medical Literature Analysis and Retrieval System Online), Embase, Scopus, and Web of Science, complemented by regulatory documents, prescribing information, FDA and European Medicines Agency (EMA) safety communications, and LiverTox. Pre-approval clinical trials first identified hepatic adverse events more frequently in avacopan-exposed patients than in comparators, although phenotypic characterization was limited. Subsequent case reports, observational cohorts, and FDA Adverse Event Reporting System (FAERS)-based pharmacovigilance analyses strengthened this concern. Recent large-scale FAERS evaluations have included more than 3,000 avacopan-related adverse event reports, including analyses of 3,529 reports and 3,150 reports in which avacopan was recorded as the primary suspect drug. Across these analyses, hepatobiliary events included abnormal liver function, elevated transaminases, jaundice, cholestasis, drug-induced liver injury (DILI), and vanishing bile duct syndrome (VBDS). Although hepatocellular and mixed patterns have been reported, the most clinically concerning phenotype is cholestatic-biliary injury, including prolonged jaundice, small bile duct injury, ductopenia, and VBDS, occasionally with fatal outcomes. Reported onset most often occurred within the first one to three months of therapy, although later presentations were also described. The FDA and EMA have responded by strengthening product safety information and recommending baseline liver assessment and close monitoring, particularly during the first six months of treatment. The mechanism remains unknown; the most plausible interpretation is multifactorial idiosyncratic DILI, potentially influenced by host susceptibility, CYP3A-related exposure, drug-drug interactions, and impaired biliary recovery in severe cases. Because causal attribution is complicated by concomitant immunosuppressive and antimicrobial therapies, avacopan should be used with careful baseline evaluation, serial liver monitoring, and prompt interruption when clinically significant liver injury is suspected.

## Introduction and background

Antineutrophil cytoplasmic antibody (ANCA)-associated vasculitis is a potentially severe, relapsing systemic autoimmune disease characterized by inflammation of small- and medium-sized vessels, with multiorgan involvement, particularly affecting the kidneys and respiratory tract. For decades, treatment has relied on high-dose glucocorticoids combined with rituximab or cyclophosphamide, regimens that are effective but associated with a substantial burden of adverse effects and toxicity. Avacopan, an oral selective antagonist of the C5a receptor (C5aR1/CD88), inhibits C5a-mediated neutrophil activation and chemotaxis and has therefore been incorporated as a strategy to reduce glucocorticoid exposure in adults with severe active ANCA-associated vasculitis [1,2].

A relevant safety signal associated with avacopan has been impaired liver function, a concern identified during its clinical development. In the pivotal ADVOCATE (Avacopan Development in Vasculitis to Obtain Corticosteroid elimination and Therapeutic Efficacy) trial [1,3] and in the pooled analysis of three clinical trials [4], avacopan was associated with elevations in liver enzymes and with a higher proportion of serious hepatic adverse events compared with non-exposed groups. In that pooled analysis, these abnormalities occurred in 4.4% of patients treated with avacopan versus 2.8% of comparators [3]. Although many cases had confounding factors and did not meet classical criteria for severe liver injury, these findings established a potential signal that warranted clinical and regulatory surveillance.

Subsequent clinical experience and pharmacovigilance further reinforced this signal. In a Japanese retrospective cohort, elevation of liver enzymes was the most frequent adverse event, observed in eight of 21 patients (38.1%), with early treatment discontinuation in a considerable proportion of cases [5]. Consistently, LiverTox considers avacopan a rare but probable cause of clinically apparent liver injury, often presenting with a cholestatic pattern [2]. More recently, on March 31, 2026, the FDA issued a safety communication regarding serious cases of drug-induced liver injury (DILI) associated with avacopan, including fatal cases and vanishing bile duct syndrome (VBDS) [6].

Large-scale FDA Adverse Event Reporting System (FAERS) disproportionality analyses have also shown that avacopan-associated adverse event reports extend beyond hepatobiliary events [7]. The most frequently reported clusters include general symptoms such as fatigue and headache; gastrointestinal disorders such as nausea, diarrhea, and abdominal discomfort; hepatobiliary events such as abnormal liver function, elevated transaminases, jaundice, cholestasis, and DILI; and infections, including serious infections and sepsis [7-9]. Within this broader postmarketing safety profile, hepatobiliary events remain particularly relevant because of their potential severity, regulatory implications, and association with cholestatic-biliary injury and VBDS.

In this context, merely describing the frequency of these events is insufficient. The clinical heterogeneity of reported cases, the concomitant use of potentially hepatotoxic therapies, and the emergence of severe cholestatic phenotypes make it necessary to review plausible hepatotoxic mechanisms and the challenges involved in causal attribution. Therefore, this review aims to synthesize the available clinical and regulatory evidence on avacopan-associated liver injury and to discuss the potential mechanisms that may explain this safety signal.

## Review

Materials and methods

This is a critical narrative review informed by a targeted literature search. The purpose was to synthesize and critically interpret the available clinical, pharmacovigilance, regulatory, and mechanistic evidence on avacopan-associated DILI, with emphasis on reported liver injury patterns, plausible mechanisms, confounding factors, and clinical implications. The narrative approach was selected because the available evidence is heterogeneous and includes clinical trials, observational cohorts, case reports, case series, pharmacovigilance studies, regulatory communications, prescribing information, and secondary literature. The review was guided by the following question: What evidence supports a potential association between avacopan and liver injury, and what mechanisms may plausibly explain this adverse event?

Information Sources and Search Strategy

Targeted searches were conducted in PubMed/MEDLINE (Medical Literature Analysis and Retrieval System Online), Embase, Scopus, and Web of Science from database inception through April 24, 2026. Searches were limited to publications in English or Spanish. Additional sources included prescribing information, FDA and European Medicines Agency (EMA) regulatory communications, and LiverTox. Reference lists of selected articles were also reviewed to identify additional relevant sources.

The search strategy combined terms related to avacopan with terms related to liver injury. The core search concept was: *(*“avacopan” OR “Tavneos”) AND (“hepatotoxicity” OR “drug-induced liver injury” OR “DILI” OR “liver injury” OR “hepatic injury” OR “cholestasis” OR “cholestatic hepatitis” OR “jaundice” OR “vanishing bile duct syndrome” OR “VBDS”). Search terms were adapted to the syntax of each database. In PubMed/MEDLINE, relevant Medical Subject Headings (MeSH) terms were considered when available, together with free-text terms.

Eligibility Criteria

Sources were considered eligible if they provided clinically or mechanistically relevant information on avacopan-associated liver injury. This included reports describing hepatic adverse events, biochemical patterns of injury, cholestatic or hepatocellular presentations, histopathological findings, latency, severity, outcomes, proposed mechanisms, risk factors, confounding medications, or recommendations for monitoring and management.

Eligible sources included clinical trials, observational studies, pharmacovigilance analyses, case reports, case series, narrative or systematic reviews relevant to the topic, prescribing information, and regulatory safety documents. Publications focused exclusively on efficacy without hepatic safety information, duplicate reports, and sources in which the relationship between avacopan and liver injury could not be meaningfully assessed were excluded.

Study Selection Process

Titles and abstracts were first screened for relevance to avacopan-associated hepatic safety. Full texts of potentially relevant sources were then reviewed by the author team, and final inclusion was based on clinical relevance, directness of evidence, and contribution to the review question. Disagreements regarding relevance or interpretation were resolved through discussion among the authors. This was not a systematic review; however, the number of initially identified records, excluded duplicates, and included sources was reported to improve transparency.

Data Charting and Narrative Extraction

Information from included sources was narratively extracted and organized according to its relevance to the review question. Extracted information included study type, population, indication for avacopan, concomitant medications, type of hepatic event, biochemical pattern of liver injury, latency, severity, histological findings when available, management, clinical outcome, and authors’ proposed interpretation.

To reduce overinterpretation, directly reported clinical findings were distinguished from hypothesis-generating elements, such as proposed mechanisms, suspected risk factors, and causal attribution. Particular attention was given to confounding by concomitant immunosuppressive or antimicrobial therapies, underlying ANCA-associated vasculitis, infection, and pre-existing liver disease.

Evidence Synthesis and Analysis

The evidence was synthesized narratively using thematic grouping. The initial analytical domains were predefined according to the review objectives and included clinical evidence of hepatotoxicity, pharmacovigilance, and regulatory evidence, reported liver injury patterns, plausible mechanisms, confounding factors, and clinical implications. These domains were refined narratively as recurrent themes emerged from the reviewed literature, particularly cholestatic-biliary injury, CYP3A-related exposure modulation, host susceptibility, and limitations in causal attribution.

No quantitative pooling was performed because of the heterogeneity of evidence sources, variability in outcome definitions, limited number of detailed clinical cases, and absence of uniform denominator-based incidence data. Pharmacovigilance findings were interpreted descriptively and cautiously, recognizing the limitations of spontaneous reporting systems, including underreporting, reporting bias, incomplete clinical information, and inability to establish individual causality.

Mechanistic framework

Mechanism of Action of Avacopan

Avacopan is an orally administered small molecule that acts as a selective antagonist of the complement C5a receptor (C5aR1/CD88). By blocking the interaction between C5a and its receptor, avacopan inhibits C5a-mediated neutrophil activation and migration. This effect is biologically relevant in ANCA-associated vasculitis, in which complement activation and neutrophil-dependent inflammatory amplification contribute to small- and medium-vessel injury. However, although its therapeutic rationale is well established, the official prescribing information states that the precise mechanism by which avacopan exerts its clinical effect in ANCA-associated vasculitis has not been fully established [2,10].

Clinical Indication and Therapeutic Context

Avacopan is indicated for adults with severe active ANCA-associated vasculitis, specifically granulomatosis with polyangiitis (GPA) and microscopic polyangiitis (MPA), as adjunctive therapy within a combination regimen. Both the United States and European regulatory documents position avacopan in combination with standard therapy based on glucocorticoids, rituximab, or cyclophosphamide, rather than as monotherapy or as a complete substitute for glucocorticoids [2,10]. This point is methodologically relevant when interpreting hepatic safety, as adverse events observed with avacopan usually occur in the setting of concomitant immunosuppression and severe systemic disease.

Pharmacokinetic Considerations Relevant to Toxicity Interpretation

The recommended dose of avacopan is 30 mg twice daily with food. Administration with food increases systemic exposure, with an approximate 72% increase in area under the curve (AUC) and an 8% increase in C_max_, with a T_max_ of two to six hours. Avacopan is more than 99.9% bound to plasma proteins, has a large apparent volume of distribution of 345 L, and has a prolonged elimination half-life of approximately 97.6 hours for the parent compound. In patients with ANCA-associated vasculitis, steady state is reached at approximately 13 weeks, with an accumulation of approximately fourfold [2,11].

Hepatic Metabolism, CYP3A4, and Drug Interactions

From a pharmacological standpoint, a key aspect of this review is that avacopan is primarily metabolized by CYP3A4. Its exposure increases substantially with strong inhibitors of this pathway; accordingly, the United States prescribing information recommends reducing the dose to 30 mg once daily when avacopan is coadministered with these agents. Conversely, strong inducers markedly decrease avacopan exposure: with rifampicin, avacopan AUC was reduced to approximately 7% of that observed in the absence of the inducer, whereas itraconazole increased exposure by slightly more than two-fold [2,11]. 

In addition, avacopan acts as a moderate inhibitor of CYP3A4 and may increase exposure to other substrates of this enzyme; in drug-drug interaction studies, it substantially increased systemic exposure to simvastatin. Therefore, CYP3A4 interaction does not by itself demonstrate a hepatotoxic mechanism, but it provides a plausible framework for understanding how increased exposure to avacopan or to concomitant medications could modulate susceptibility to liver injury in certain patients [2,11].

Patient-Related Confounding Factors in ANCA-Associated Vasculitis

The assessment of hepatic safety in ANCA-associated vasculitis is particularly complex because the typical patient may have multiple potential sources of liver test abnormalities. Patients in the pivotal studies received rituximab or cyclophosphamide, and glucocorticoids were permitted according to clinical need. Additional factors include intercurrent infections, antimicrobial prophylaxis, viral reactivation, particularly hepatitis B, for which screening is recommended before treatment initiation, multiorgan involvement, and nonspecific elevations in liver biochemical tests in the setting of severe systemic inflammation. Therefore, in this disease, causal attribution of DILI can rarely be based on temporality alone and requires an explicit differential assessment against concomitant immunosuppressive agents, pre-existing liver disease, viral infection, and underlying disease activity [6,10,11].

Evidence of hepatotoxicity

Clinical Trials and Pre-Approval Data

In the pivotal ADVOCATE trial [3] and in pre-approval safety analyses, hepatic abnormalities were mainly described as elevations in liver enzymes and hepatic adverse events requiring specific clinical follow-up. Although the phenotypic characterization of liver injury was limited and many patients were receiving potentially hepatotoxic concomitant therapies, these findings prevented the signal from being considered an isolated or incidental event [1,4].

This observation was reinforced by the pooled analysis of three clinical trials [1,3,12,13], in which events related to hepatic abnormalities were more frequent among patients treated with avacopan than among comparators. In that analysis, these abnormalities occurred in 4.4% of patients exposed to avacopan versus 2.8% of those not exposed, suggesting that the hepatic safety signal was already present during the premarketing phase [4]. Rather than establishing a defined mechanism or an unequivocal biochemical pattern, these data demonstrated a relative increase in hepatic events and supported closer monitoring during the subsequent development of the drug. 

The pre-approval evidence did not allow a precise determination of whether the predominant injury phenotype was hepatocellular, cholestatic, or mixed. Nor did it consistently provide the clinical details that would later be reported in case reports and postmarketing cohorts regarding latency, jaundice, rechallenge, or histology. In this regard, the trial data were more useful for identifying a quantitative signal of hepatic risk than for defining a specific clinicopathological phenotype [1,4].

Furthermore, causal interpretation of the events observed in the trials was limited by confounding factors, including the complexity of severe active ANCA-associated vasculitis and the concomitant use of other medications. Therefore, although pre-approval data did not conclusively demonstrate DILI with a uniform pattern, they did establish a sufficiently consistent safety alert to support subsequent regulatory surveillance and the need for more precise clinical characterization in real-world practice [4].

Regulatory Safety Signal

In Europe, the EMA maintains that the benefit-risk balance of Tavneos® (avacopan) remains favorable, but recognizes liver function abnormalities as among the most clinically relevant serious adverse events and includes explicit risk-minimization measures in the product information [10]. The updated summary of product characteristics warns of severe transaminase elevations accompanied by increased total bilirubin and, in the postmarketing setting, cases of DILI and VBDS, including fatal outcomes. Accordingly, it recommends baseline testing of transaminases and total bilirubin, avoiding treatment initiation in patients with liver disease or liver tests >3 times the upper limit of normal, and monitoring at least every four weeks during the first six months of treatment [10]. 

The FDA reinforced this concern in its Drug Safety Communication of March 31, 2026, warning of serious postmarketing cases of DILI associated with avacopan, including fatal cases and cases complicated by VBDS [6]. The agency noted that, although hepatotoxicity was already part of the premarketing safety profile, cases of VBDS and fatal DILI represented new safety concerns. After reviewing the marketing authorization holder’s global database, the literature, and FAERS/AEMS through October 9, 2024, the FDA identified 76 cases of DILI with reasonable evidence of a causal association; 74 were serious, including 54 hospitalizations and 8 deaths. Among cases with sufficient laboratory data, a cholestatic or mixed pattern predominated, with a median time to onset of 46 days, and seven biopsy-confirmed cases of VBDS were identified, three of which were fatal [6]. 

Taken together, the FDA and EMA agree that avacopan-associated hepatotoxicity represents a clinically relevant regulatory safety signal, more clearly defined by postmarketing experience. The response of both agencies has not been to withdraw the drug, but rather to strengthen labeling, intensify hepatic monitoring, and establish a lower threshold for suspecting and discontinuing treatment when evidence of DILI or cholestasis emerges. This regulatory evolution supports the interpretation that potential avacopan-associated hepatotoxicity should not be understood merely as nonspecific liver enzyme abnormalities, but as a potential risk of severe hepatobiliary injury, particularly with a cholestatic phenotype [6,10,14].

Case Reports and Case Series

The included case reports and case series show that liver injury associated with avacopan may occur from a few weeks to several months after treatment initiation, with a temporal clustering during the early phase of exposure. In individual cases, latency ranged from 31 days to approximately three months [15-21]; in a Japanese multicenter cohort, it ranged from 25 to 189 days, with a median of 54 days [22]; and in another Japanese cohort, the median was approximately 1.7 months [5]. This temporal pattern is compatible with idiosyncratic DILI, although it does not by itself establish causal attribution. 

From a phenotypic standpoint, the most consistent finding in the best-characterized cases was cholestatic or biliary involvement [16-18,20-22]. Acute cholestatic hepatitis, prolonged cholestasis, small bile duct injury, and probable or confirmed VBDS were described, including a positive rechallenge case [17] and fatal cases [20,22]. However, the spectrum was not uniform: one case was formally classified as hepatocellular, with an R ratio of 11.5 [19], and a multicenter series identified hepatocellular, cholestatic, and mixed episodes [20]. This indicates that the hepatic safety signal associated with avacopan is clinically heterogeneous and should not be reduced to a single phenotype.

The most persuasive case in terms of chronology and causal attribution is that reported by Tang et al., in which an initial exposure was discontinued because of rash after two days, and subsequent reintroduction was associated with severe hyperbilirubinemia and a cholestatic-biliary presentation compatible with probable VBDS 51 days later [17]. Nevertheless, relevant confounders remained, including prior DILI and concomitant exposure to other medications. By contrast, Mori et al. reported reintroduction of avacopan without recurrence of DILI in four patients, suggesting that susceptibility is not uniform [20]. Therefore, a negative rechallenge does not invalidate the hepatotoxic signal, but rather highlights the biologically variable expression of the event.

Clinical severity was also variable. Some reports documented isolated aminotransferase elevations without hyperbilirubinemia, with rapid resolution after treatment discontinuation or dose reduction, whereas others progressed with marked jaundice, pruritus, a prolonged course lasting several months, or death due to liver failure associated with VBDS [5,18,20,22,23]. Overall, evidence from case reports and case series supports a genuine clinical signal, although causal attribution should be formulated cautiously because of the frequent simultaneous exposure to rituximab, trimethoprim-sulfamethoxazole, cyclophosphamide, glucocorticoids, and other potentially hepatotoxic agents.

Pharmacovigilance/Postmarketing Evidence

The available postmarketing evidence further supports a potential hepatobiliary toxicity concern associated with avacopan. The three included studies, all based on FAERS data, identified a higher-than-expected disproportionality of hepatic reports, although they differed in the analysis period, the magnitude of the estimates, and the statistical approach used [7-9].

Consistently, the signal was not limited to nonspecific biochemical abnormalities. The analyses identified terms related to hepatobiliary disorders, abnormal liver function, DILI, jaundice, and cholestasis. In addition, two studies detected a signal for VBDS, a finding of particular relevance because of its potential severity and its concordance with recent regulatory warnings [7,8]. This recurrence across independent analyses suggests that the hepatic signal associated with avacopan has a clinically recognizable hepatobiliary expression beyond isolated transaminase elevations. 

The magnitude of disproportionality varied, but it was reproducible for events with greater clinical specificity. Yang et al. reported signals for hepatobiliary disorders, abnormal liver function, DILI, jaundice, cholestasis, and VBDS, with particularly high odds ratios for VBDS and suspected DILI [7]. Li et al., using a more conservative analysis, also identified signals for hepatobiliary disorders, increased liver enzymes, hepatic disorder, abnormal liver function, elevated liver function tests, and DILI [9]. Similarly, Chang et al. confirmed signals for hepatic disorder, jaundice, abnormal liver function, DILI, cholestasis, and VBDS. Although the point estimates should be interpreted with caution, the repeated pattern strengthens the plausibility of a genuine hepatobiliary safety signal [8].

The timing of events was also relatively consistent. Yang et al. reported a median time to onset of 48 days [7], Chang et al. of 55.5 days [8], and Li et al. of 86.5 days [9]. Across the three studies, a substantial proportion of events occurred within the first month or the first two months of treatment, although late-onset cases were also described. This pattern is compatible with predominantly early-onset hepatic toxicity, without excluding delayed presentations, and supports the need for close monitoring during the first months of exposure.

The severity of the reported events was clinically relevant. Yang et al. reported hospitalization in 23.3% and death in 7.5% of reports [7]; Li et al. described initial or prolonged hospitalization in 19.5% and death in 6.9% [9]; and Chang et al. reported hospitalization in 15.98% and death in 5.59% [8]. These proportions should not be interpreted as true incidence rates, given the spontaneous nature of FAERS reporting and the lack of denominators, but they indicate that the postmarketing signal includes serious events and not merely mild laboratory abnormalities. 

Some findings suggest potential risk modifiers, although they do not allow definitive conclusions. Yang et al. observed a higher likelihood of hepatobiliary events in older patients, particularly those aged ≥65 years, as well as associations with female sex, rituximab, and proton pump inhibitors [7]. Li et al. described a higher frequency of hepatic dysfunction among Japanese patients compared with American or Caucasian patients [9], whereas Chang et al. identified differences by sex and age for specific events, although less consistently [8]. Taken together, these data suggest that susceptibility to hepatotoxicity may be influenced by age, polypharmacy, therapeutic context, and individual patient characteristics.

In summary, pharmacovigilance studies provide a reproducible hepatobiliary signal, with convergence toward clinically specific events such as DILI, cholestasis, jaundice, and VBDS. Nevertheless, these findings should be interpreted in light of the inherent limitations of FAERS, including underreporting, reporting bias, absence of denominators, incomplete clinical information, and difficulty in establishing causality at the individual level.

Reported patterns of liver injury

Hepatocellular Phenotype

A hepatocellular phenotype was explicitly documented by Yamashita et al., who classified the event as hepatocellular with an R ratio of 11.5 and described marked elevations in aspartate aminotransferase (AST) and alanine transaminase (ALT) accompanied by progressive hyperbilirubinemia [19]. The pediatric case reported by Nishino et al. also showed an abrupt and predominant increase in aminotransferases, compatible with a hepatocellular or mixed pattern, although no formal classification was provided [15]. The series by Mori et al. further confirmed that the spectrum associated with avacopan is not restricted to cholestasis, as it included hepatocellular episodes defined by the R ratio within a phenotypically variable set of cases [20].

Cholestatic Phenotype and Biliary Injury

The most consistent pattern among clinically informative reports, however, was cholestatic or biliary. Yamaguchi et al. described VBDS with severe jaundice and persistence of hyperbilirubinemia for five months [16]. Tang et al. reported acute cholestatic hepatitis with small bile duct injury and probable VBDS after rechallenge [17]. Kioi et al. documented acute cholestasis with prolonged jaundice and accumulation of lipoprotein X, a finding secondary to severe cholestasis [18]. Kojima et al. described prolonged jaundice with biopsy findings compatible with centrilobular cholestasis and focal necrosis [21]. Finally, both Mori et al. [20] and Hishinuma et al. [22] provided the most compelling evidence of severe biliary injury: the former through a multicenter cohort that included a fatal case of VBDS, and the latter through a fatal case with persistent ductopenia confirmed by biopsy and autopsy.

Mixed Phenotype and Heterogeneity

The main difficulty in synthesizing liver injury patterns is that several articles did not explicitly report whether the injury was hepatocellular, cholestatic, or mixed. Therefore, for a methodologically robust interpretation, it is important to distinguish between the formal classification provided by the authors and the predominant phenotype that can be inferred from chronology, biochemical profile, bilirubin levels, and histology. Under this approach, the evidence suggests a predominance of cholestatic-biliary injury, but within a continuum that also includes hepatocellular and mixed forms [15,19,20].

Jaundice, Chronology, Severity, and Reversibility

Jaundice or clinically relevant hyperbilirubinemia appeared in multiple reports and was consistently associated with greater severity and a more prolonged clinical course [16-23]. Overall, the chronology suggests that most events occur within the first 4-12 weeks of treatment, although the multicenter series showed that the range may extend to almost six months [5,15-22]. In terms of severity, the spectrum was broad, ranging from reversible enzyme elevations without increased bilirubin to severe jaundice, persistent cholestasis, probable bile duct injury, and death [5,20,22,23]. Reversibility was therefore not uniform. Many patients improved after avacopan discontinuation, but some experienced slow recovery over weeks or months, and a subgroup progressed to severe biliary injury with an irreversible outcome [16,18,20-22].

In addition, a recent editorial focused on the Japanese experience emphasized that cases of DILI with marked bilirubin elevation or VBDS tended to occur within the first month after avacopan initiation [24]. Although this observation derives from an interpretative synthesis rather than from a comparative cohort designed to estimate risk, it is consistent with the impression that the most severe cholestatic-biliary subphenotype may appear early and follow a disproportionately severe course in selected patients.

The potential mechanism of hepatotoxicity

Idiosyncratic Immune-Mediated Injury

Based on the current evidence, the most defensible hypothesis is that of idiosyncratic DILI, possibly immune-mediated, although this has not been established. Several findings are compatible with this interpretation: the absence of a clearly dose-dependent pattern, a latency of several weeks compatible with sensitization, the occurrence of rash during initial exposure, severe recurrence after rechallenge in one case, portal or eosinophilic inflammatory infiltrates in some biopsies, and clinical response to mycophenolate mofetil in one case refractory to drug withdrawal alone [17,19,21,22]. However, these findings remain indirect. No specific mechanistic demonstration exists, immunological studies have been limited, and lymphocyte stimulation tests, when performed, were negative [15,22]. Therefore, it is more rigorous not to refer to an established immunological mechanism, but rather to a biologically plausible possibility within the spectrum of idiosyncratic DILI.

Role of Hepatic Metabolism and CYP3A4

Among the included articles, the relationship with CYP3A4 appears mainly as a hypothesis of susceptibility or altered systemic exposure, rather than as a proven causal mechanism. Some reports mention CYP3A4/CYP3A5 polymorphisms, possible metabolic competition with other drugs, and the potential contribution of drug-drug interactions, including concomitant use of agents metabolized through the same pathway or drugs such as vonoprazan [7,16,20,22]. However, none of the studies provided clinical pharmacokinetic data, plasma concentrations of avacopan, or an exposure-response gradient that would robustly link increased exposure to greater hepatotoxicity. Consequently, CYP3A4 should be discussed in the manuscript as a plausible modulator of risk or severity, but not as a demonstrated mechanism of liver injury.

Hishida and Nagata's editorial adds a pharmacogenetic dimension to this hypothesis by noting that, in the Japanese population, the high frequency of the CYP3A5*3 allele and *3/*3 homozygosity could shift avacopan metabolism further toward CYP3A4 [24]. In this context, the coexistence of CYP3A4 inhibitors or inducers and the polypharmacy inherent to the treatment of ANCA-associated vasculitis could amplify systemic exposure or modify susceptibility to liver injury. However, this interpretation remains indirect, as no robust clinical link has yet been demonstrated between CYP3A genotype, avacopan concentrations, and DILI severity. 

Cholestatic Phenotype and Potential Biliary Injury

Among the available mechanistic hypotheses, the one most strongly supported by clinical and histopathological phenomenology is that of a cholestatic-biliary subphenotype. Several studies described marked hyperbilirubinemia, pruritus, prolonged jaundice, small bile duct injury, progressive ductopenia, and probable or confirmed VBDS [16-18,20-22]. The histological convergence is particularly notable: mild or mixed portal inflammatory infiltrates, diffuse cholestasis, bile duct injury or loss, and absence of biliary regeneration in the most severe cases [16,20,22]. This recurring ductopenic phenotype, including cases with fatal outcomes, suggests that the hepatotoxic signal associated with avacopan is not merely a nonspecific elevation of transaminases, but may acquire a clinically significant biliary signature in a subset of patients. 

A particularly useful contribution of the editorial by Hishida and Nagata is the proposal that pharmacological blockade of C5aR may not only participate in the initial injury, but also impair hepatic and biliary regeneration [24]. They support this possibility with animal studies in which C5aR deficiency was associated with greater toxic liver injury, reduced compensatory hepatocyte proliferation, and increased apoptosis. Although this extrapolation does not demonstrate a specific human mechanism for avacopan, it provides a biologically plausible framework for interpreting the persistent ductopenia, prolonged cholestasis, and VBDS observed in the most severe cases.

Alternative Diagnoses and Confounders

This subsection is likely, together with the cholestatic phenotype, one of the strongest points of the discussion. Across nearly the entire evidence base, there was concomitant exposure to one or more drugs with hepatotoxic potential, particularly rituximab, trimethoprim-sulfamethoxazole, cyclophosphamide, glucocorticoids, amoxicillin-clavulanate, and azithromycin [5,15-22,25]. In addition, ANCA-associated vasculitis itself, viral infection, and other immune-mediated or obstructive liver diseases are part of the differential diagnosis. 

Some articles strengthened causal attribution to avacopan by serologically excluding viral hepatitis, ruling out obstructive cholestasis on imaging, or demonstrating biochemical improvement after avacopan discontinuation despite continuation of other therapies [5,17,19,21]. However, causality assessment tools, including RUCAM (Roussel Uclaf Causality Assessment Method) [26]**, **Naranjo scale [27]**, **and Revised Electronic Causality Assessment Method for Japan (RECAM-J) [28], showed relevant limitations when several drugs were initiated in parallel, and in some cases, the score was similar for avacopan and other agents [20-22]. Therefore, a mature interpretation should acknowledge that the signal exists, while recognizing that individual causality remains incomplete in a substantial proportion of cases.

Host Factors

Host factors should be presented as exploratory signals rather than established predictors. Across the studies, recurrent factors included advanced age, polypharmacy, possible genetic or ethnic susceptibility among Japanese patients, a prior history of DILI, and, in the multicenter series, an association between greater severity and lower body mass index [5,17-20,22,25]. These observations are plausible and clinically suggestive, but they do not yet allow definition of a robust risk profile, partly because pre-existing liver disease, genotype, and pharmacokinetic exposure were not systematically assessed. Accordingly, the manuscript may propose that these factors modulate susceptibility, provided that it explicitly states that the evidence remains insufficient to establish them as causal determinants. 

Hishida and Nagata’s editorial characterized host risk factors for severe DILI in Japanese cohorts [24]. They identified advanced age, low body mass index, an myeloperoxidase (MPO)-ANCA-positive/microscopic polyangiitis phenotype, and early-onset hepatic dysfunction as factors associated with severe outcomes, including marked hyperbilirubinemia and VBDS. However, these factors should be interpreted as exploratory susceptibility signals rather than established predictors, because the available evidence derives from small cohorts with substantial pharmacological confounding and limited external validation.

Integrative Pathophysiological Hypothesis

The available evidence does not allow a single established pathophysiological mechanism to be proposed for avacopan-associated hepatotoxicity. However, integration of the clinical, histopathological, regulatory, and pharmacovigilance findings supports a plausible model of multifactorial idiosyncratic liver injury, rather than intrinsic dose-dependent toxicity. In this model, baseline host susceptibility, polypharmacy, and modulation of drug exposure may favor the development of DILI in a subset of patients with ANCA-associated vasculitis, particularly in contexts of advanced age, low body mass index, possible genetic predisposition, and concomitant treatment with other potentially hepatotoxic agents.

**Figure 1:**
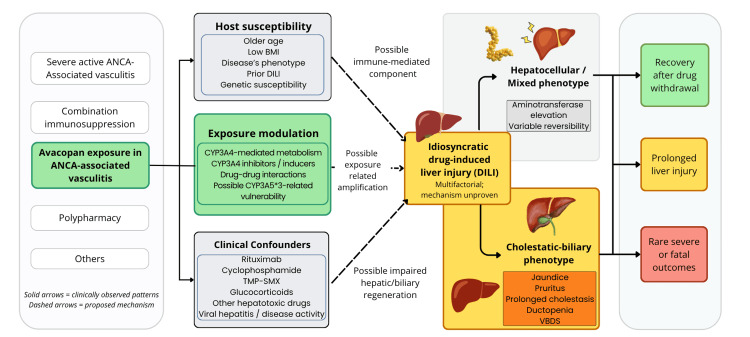
Proposed integrative pathophysiological model of avacopan-associated liver injury. Current evidence supports a multifactorial idiosyncratic DILI model in which host susceptibility, CYP3A4-related exposure modulation, drug-drug interactions, and clinical confounders may facilitate liver injury after avacopan exposure. Although hepatocellular and mixed patterns have been described, the most clinically concerning phenotype appears to be cholestatic-biliary, including ductopenia and VBDS. The mechanistic links remain hypothetical and should not be interpreted as established causal pathways. DILI: drug-induced liver injury; VBDS: vanishing bile duct syndrome; ANCA: antineutrophil cytoplasmic antibodies; TMP-SMX: trimethoprim-sulfamethoxazole Image Credit: Authors; created using Canva (Canva Pty Ltd., Sydney, Australia) and does not contain generative AI-generated content.

Within this framework, CYP3A4 should be interpreted as a plausible modulator of risk or severity, rather than as an established causal mechanism. In parallel, the recurrence of marked hyperbilirubinemia, prolonged cholestasis, ductal injury, ductopenia, and VBDS suggests that, in some patients, avacopan-associated injury adopts a cholestatic-biliary phenotype of particular clinical relevance. In this context, the hypothesis that C5aR blockade may interfere with hepatic and biliary regeneration provides an attractive biological framework for interpreting the persistence of cholestasis and progression to VBDS, although this possibility remains indirect and has not been demonstrated in humans. Overall, the most cautious model is that of avacopan-associated idiosyncratic DILI, modulated by host susceptibility, drug-drug interactions, and, in the most severe cases, a cholestatic-biliary expression with insufficient ductal repair. A representative example of this can be seen in Figure 1.

Clinical implications

The available evidence supports the systematic integration of hepatic monitoring into the clinical use of avacopan in ANCA-associated vasculitis. This recommendation is based not only on nonspecific elevations in liver enzymes observed in clinical trials, but also on the subsequent convergence of case reports, postmarketing cohorts, pharmacovigilance analyses, and regulatory warnings, which have demonstrated a potentially serious hepatobiliary safety signal, particularly with a cholestatic-biliary phenotype.

In clinical practice, before treatment initiation, at least AST, ALT, alkaline phosphatase, and total bilirubin should be documented, together with a baseline assessment focused on pre-existing liver disease, viral hepatitis, potential drug-drug interactions, and concomitant exposure to other potentially hepatotoxic agents. Given that most clinically relevant events appear to cluster within the first weeks or months after initiation, monitoring should be more intensive early in the course of treatment, with a low threshold for reassessment in the presence of jaundice, pruritus, hyperbilirubinemia, or an emerging cholestatic pattern.

Suspected DILI should prompt immediate discontinuation of the drug and a structured etiological evaluation. In patients with ANCA-associated vasculitis, in whom polypharmacy and alternative diagnoses are common, interpretation of liver test abnormalities requires active exclusion of viral infection, obstructive cholestasis, underlying disease activity, and hepatotoxicity from concomitant therapies. In this context, the main clinical implication of the current evidence is not the definition of a closed algorithm, but rather the need for active clinical pharmacovigilance, serial monitoring, and early intervention when findings compatible with clinically significant hepatobiliary injury are identified.

Limitations of the evidence

The evidence on avacopan-associated hepatotoxicity remains limited and methodologically heterogeneous. This review integrates clinical trials, observational cohorts, case reports, pharmacovigilance analyses, and regulatory documents; however, the diversity of study designs, objectives, and levels of detail precludes direct comparison across sources and justifies the use of a qualitative rather than quantitative synthesis. Accordingly, the conclusions should be interpreted as a critical integration of an incomplete evidence base, rather than as a definitive estimate of hepatotoxic risk.

A central limitation is that pre-approval data identified a hepatic safety signal but did not characterize the clinical phenotype of liver injury with sufficient precision. Current understanding of the pattern of injury, latency, severity, and reversibility relies largely on small series and individual case reports, in which descriptive detail is greater but causal attribution is more vulnerable to bias and confounding. This is further compounded by the frequent simultaneous exposure to rituximab, cyclophosphamide, glucocorticoids, trimethoprim-sulfamethoxazole, and other potentially hepatotoxic agents, which limits individual attribution to avacopan even when the chronology is suggestive.

Pharmacovigilance analyses, in turn, strengthen the external consistency of the signal, but share the inherent limitations of spontaneous reporting systems, including underreporting, duplicate reports, notoriety bias, variable data quality, and the absence of reliable denominators. Important mechanistic gaps also remain, including the lack of specific biomarkers, systematic clinical pharmacokinetic data, and external validation of potential host susceptibility factors. Finally, the most severe and best-characterized cases come largely from Japanese cohorts, which requires consideration of possible geographic bias, population-level differences, or variation in the intensity of monitoring and publication practices.

## Conclusions

The available evidence supports a potential avacopan-associated hepatotoxicity. This concern emerged during clinical development, was reinforced by postmarketing experience, and has been recognized by regulatory agencies. Although the clinical spectrum is not uniform, avacopan-associated liver injury should no longer be interpreted merely as a marginal laboratory abnormality, but rather as a potentially clinically relevant adverse event. From a phenotypic standpoint, the currently available evidence suggests a relative predominance of cholestatic or biliary forms, including presentations with marked jaundice, ductal injury, progressive ductopenia, and VBDS. However, hepatocellular and mixed forms have also been described; therefore, avacopan-associated hepatotoxicity should be conceptualized as a clinical continuum in which a subset of patients appears to progress toward disproportionately severe cholestatic-biliary injury.

The underlying mechanism remains to be established. In light of the current evidence, the most reasonable hypothesis is that of idiosyncratic DILI, probably modulated by host susceptibility, drug-drug interactions, therapeutic confounding, and potential interference with hepatic or biliary recovery in severe cholestatic cases. Consequently, the central clinical message is twofold: potential hepatotoxicity associated with avacopan should be recognized, but its interpretation requires causal caution, serial hepatic monitoring, and active differential evaluation in patients with ANCA-associated vasculitis treated with avacopan.
